# The Evolving Landscape of Vision Rehabilitation: Current Status and Future Directions

**DOI:** 10.1007/s44402-026-00088-2

**Published:** 2026-05-04

**Authors:** Amritha Stalin, Andrew Miller, Hilde van der Aa, Simona Turco, Tammy Labreche, Stefania Fortini, Carlotta Lalla, Filippo Amore, Michael Crossland, Gianni Virgili, Benjamin Thompson, Ruth van Nispen

**Affiliations:** 1 School of Optometry and Vision Science, University of Waterloo, Waterloo, Ontario Canada; 2 Centre for Eye and Vision Research, Hong Kong, Hong Kong; 3 School of Psychology, Health and Clinical Sciences, Aston University, Birmingham, UK; 4 Amsterdam UMC, Vrije Universiteit Amsterdam, Department of Ophthalmology, Amsterdam Public Health research institute, Amsterdam, The Netherlands; 5 National Centre of Services and Research for the Prevention of Blindness and Vision Rehabilitation of the Visually Impaired-IAPB ETS, Fondazione Policlinico Universitario A. Gemelli, IRCCS, Rome, Italy; 6 Moorfields Eye Hospital NHS Foundation Trust, London, UK; 7 UCL Institute of Ophthalmology, London, UK; 8 NEUROFARBA, University of Florence, Florence, Italy

**Keywords:** Mental health, Multidisciplinary rehabilitation, Neuroplasticity, Technology, Visual impairment, Vision rehabilitation

## Abstract

Vision impairment is increasingly recognised as a complex condition shaped not only by ocular pathology but also by cognitive, psychological, social and environmental factors that influence participation and quality of life. In line with the United Nations Convention on the Rights of Persons with Disabilities and the International Classification of Functioning, Disability and Health, contemporary vision rehabilitation frameworks adopt a biopsychosocial perspective that extends beyond treatment of the eye condition alone toward holistic support to improve the lives of individuals with vision loss. An overview of the effectiveness and future directions of multidisciplinary vision rehabilitation in clinical practice is presented, and a summary of the findings concerning the international standards of vision rehabilitation, focusing on adults. Furthermore, some examples of achievements in emerging fields are presented, such as neuroplasticity and visual system recovery, technological interventions and psychosocial support, including their future directions. Multidisciplinary models are widely supported but remain difficult to implement because of limited resources, workforce constraints and differences in culture and health policy. Overcoming these barriers is critical to expanding and strengthening multidisciplinary vision rehabilitation. The evidence highlights the need for implementation-focused research, closer collaboration across disciplines and core outcome measures that capture participation, mental health and quality of life, not just impairment. The overview also points to the importance of structured models that integrate neurorehabilitation (artificial intelligence-based) technology and mental healthcare while adapting to regional and cultural contexts. Embedding multidisciplinary vision rehabilitation within health systems is both a clinical necessity and a prerequisite for advancing global commitments to inclusion and equity for people with vision impairment. As population ageing and global demographic change are expected to increase the absolute numbers of adults with vision loss, strengthening collaboration between research, clinical practice and service delivery will be essential to further improve the quality of life of individuals with vision loss.

Key Points

- Vision rehabilitation is recommended to be holistic, addressing not only functional vision loss but also cognitive, psychological, social and environmental factors that shape daily functioning and quality of life.
- Multidisciplinary care is essential yet under-implemented, with progress limited by resource constraints, workforce shortages and policy differences across the globe; stronger cross-disciplinary collaboration and core outcome measures are needed.
- Future advancement depends on integrating neurorehabilitation, emerging technologies (including artificial intelligence) and mental healthcare into structured models embedded within health systems, especially as ageing populations increase the number of adults with vision loss.

## Introduction

The United Nations Convention on the Rights of Persons with Disabilities represents a paradigm shift in framing disability as a human rights issue [1]. Since its adoption in 2006, it has advanced the expectation of an inclusive society in which persons with disabilities can exercise their rights on an equal basis with others. The Convention operationalises this vision through commitments to accessibility, independent living, equal access to education, employment and healthcare and enhanced participation in social and civic life. With widespread ratification, countries are obligated to promote inclusion and equal opportunities. The Convention is grounded in the social model of disability, which conceptualises disability as arising primarily from societal, environmental and attitudinal barriers rather than from individual impairment. In turn, vision rehabilitation services, particularly in high-income contexts, typically employ biopsychosocial frameworks that integrate biological, psychological and social determinants of functioning. These perspectives reinforce one another rather than compete; the biopsychosocial model provides an operational framework for understanding and addressing vision disability in everyday life, which in turn supports the Convention’s rights-based aims by systematically identifying the contextual factors that enable or restrict participation.

A prominent example of a biopsychosocial model is the World Health Organization’s (WHO) International Classification of Functioning, Disability and Health (ICF), introduced in 2001 and widely applied in vision rehabilitation [2]. The ICF reconceptualises disability by emphasising participation and the interaction between health conditions and contextual factors. It offers a comprehensive structure in vision rehabilitation for understanding how eye diseases relate to visual functioning, activities and participation, environmental facilitators or barriers, thereby aligning clinical practice with the broader societal commitments articulated in the Convention.

To address the many consequences on the lives of people with congenital, hereditary or acquired vision loss and following the ICF, the Convention and its global societal demands, vision rehabilitation has substantially evolved over the past two decades. It has transitioned from being focused on optical aids and compensatory strategies to an individual-centred, comprehensive process that addresses various dimensions of vision impairment—functional, neurological, physical, psychological, technological and social [3–5]. Vision impairment is not just a sensory problem, but significantly impacts cognition, mobility, employment, emotional well-being, daily activities, participation in society and overall quality of life [6]. Therefore, effective vision rehabilitation increasingly requires collaboration across multiple disciplines, including optometry, ophthalmology, neuroscience, occupational therapy, education, psychology, social work, engineering and public health, among others.

Alongside monodisciplinary conferences that deepen expertise on specific themes (e.g., vision and driving or single-condition meetings such as those on hemianopia or age-related macular degeneration), the International Society for Low Vision Research and Rehabilitation (ISLRR) has played a unifying role in the field of vision rehabilitation. Since its first official ‘Vision’ meeting in the Netherlands in 1993, it has brought together clinicians, researchers and innovators working across diverse areas of low vision (from any cause) for many years, to showcase their research or their vision rehabilitation models [7]. Another example is the Low Vision section within the Association for Research in Vision and Ophthalmology annual research meetings, mainly based in the United States of America. These conferences have provided a platform for professionals from traditionally distinct disciplines to share research, clinical experiences and emerging technologies [8]. At the same time, they have strengthened interdisciplinary collaboration through the integration of knowledge, but mainly multidisciplinary collaboration, where experts work together while maintaining their distinct professional perspectives around the person with vision loss.

However, at the systems level, translating either inter- or multidisciplinary vision rehabilitation from concept to practice presents challenges. Variations in resource availability, workforce capacity, cultural context and health policy, both within and across countries, shape how services are organised and delivered [9–11]. Despite increasing global recognition of vision rehabilitation as a core component of eye health, substantial disparities persist in access to and availability of vision rehabilitation services. These challenges have been addressed by the *National Low Vision Centre* in Rome (*WHO Collaborating Centre, WHOCC ITA100)*, which has coordinated international efforts to define standards for vision rehabilitation in varied settings [12].

This paper presents an overview of the effectiveness of multidisciplinary collaboration in clinical practice and a summary of the findings of the collaboration on the international standards of vision rehabilitation, focused mainly on adults. Furthermore, it presents some examples of achievements in emerging fields, such as neuroplasticity and visual system recovery, technological interventions and psychosocial support. It also focuses on the future directions of vision rehabilitation, including these emerging fields. By exploring perspectives across these areas, we aim to highlight the opportunities and challenges present in delivering individual-centred, comprehensive vision rehabilitation in an interconnected healthcare landscape.

## Multidisciplinary Collaborations in Clinical Practice

Multidisciplinary working within low vision services has been proposed by many as a model that effectively meets the complex rehabilitation needs of people with vision impairment [9, 10, 13, 14]. In response to rising levels of vision impairment, WHO guidance has endorsed this approach, advocating for integrated, individual-centred eye care and coordinated multidisciplinary approaches to the delivery of comprehensive eye health services, including vision rehabilitation [15].

There is good evidence to suggest that collaborative approaches in vision rehabilitation lead to sustained improvements in visual ability and reduce the impact of vision loss compared to baseline [14, 16–20] and waitlist controls [13]. Despite this, findings are more equivocal for broader outcomes, with multidisciplinary approaches showing no effect on health-related quality of life and offering no additional improvement in vision-related quality of life compared to other service methods [20]. Therefore, these findings question whether current collaborative models adequately address elements of the psychological and well-being dimensions of sight loss.

Evidence directly comparing different models of vision rehabilitation is limited and largely drawn from older studies in high-income countries (HICs). One such study demonstrated that a multidisciplinary inpatient programme delivered greater improvements in visual ability and mobility compared with a cheaper community-based scheme [21]. However, beyond such examples, contemporary data comparing collaborative services directly with alternative approaches remains scarce. Therefore, future service models should be established using robust, contemporary research that reflects the evolving needs of their users.

Global rehabilitation resources are currently distributed inequitably. In many low-income countries, vision rehabilitation services are entirely absent, or where they exist, they tend to be clinically focused with a lack of input from rehabilitation officers, specialist teachers or community workers [22]. In contrast, HICs such as those in Scandinavia and Canada aim to provide well-funded services with low staff-to-patient ratios, which are better equipped to deliver a diverse range of collaborative services tailored to individual needs [9, 11].

To address resource constraints, triage models have been proposed, prioritising multidisciplinary input for individuals with the greatest identified need [11]. Additionally, ‘task-shifting’ protocols have been implemented successfully to build capacity in other areas of medicine. These strategies reallocate responsibilities from small numbers of highly specialised professionals to larger volumes of trained community workers in resource-limited settings [23]. Similar strategies could establish collaborative rehabilitation service models that adapt to local needs while incorporating diverse input, making them more responsive to the requirements of local populations. However, success within task-shifting depends on critical foundations, including training, shared purpose, defined protocols and sustainable funding [24]. Meeting these requirements would necessitate a change from current practice, where these elements may be absent from all but the most developed service models.

## International Standards of Vision Rehabilitation: Global and Cultural Perspectives

One of the first actions in 2014 of the *National Low Vision Centre* in Rome, while coordinating international efforts to define standards for vision rehabilitation in varied settings, was to prepare an analysis of Strengths-Weaknesses-Opportunities-Threats of vision rehabilitation for the six WHO regions [12]. Results gathered from the period between 2015 and 2025 have shown that the situation remained quite similar. There are scarce epidemiological data, few services and barriers to access, limited recognition of the importance of vision rehabilitation among national governments and eye care professionals, few trained personnel and low availability and high cost of assistive devices. However, the multidisciplinary model of care has been fully recognised as valid, although networks between professionals are underdeveloped [25].

Moreover, the global landscape of vision rehabilitation services changed considerably due to population ageing, increasing prevalence of diabetes and myopia and improved sight-saving treatments for previously blinding conditions [26]. At the same time, international initiatives such as the *WHO Global Action Plan for Universal Eye Health and* the *Rehabilitation 2030* initiative increased policy attention to vision rehabilitation as a core health service [27].

During the last decade, global recognition of rehabilitation as a core component of health systems has increased significantly. The *WHO Rehabilitation 2030* initiative and the *World Report on Vision* explicitly identified vision rehabilitation as essential to achieve universal eye health and *Universal Health Coverage* [15]. However, integration of vision rehabilitation into national eye-health strategies and financing mechanisms remained inconsistent across countries. At the same time, rapid advances in digital technologies and artificial intelligence (AI) expanded options in vision rehabilitation, while the COVID-19 pandemic accelerated adoption of telehealth and remote care models [28].

### Access to Vision Rehabilitation Services

In HICs, access to vision rehabilitation services has generally improved modestly during the last decade, particularly in urban areas [29]. Nevertheless, access barriers seem to persist. Rural communities, economically disadvantaged populations and older adults have continued to face significant challenges, including transportation barriers, long waiting times and fragmented referral pathways between ophthalmologists and rehabilitation services [30].

In low- and middle-income countries (LMICs), access to vision rehabilitation services has remained severely limited throughout the decade. Services frequently remained concentrated in tertiary or urban centres, with large rural populations underserved. Shortages of trained personnel, limited awareness among health professionals and lack of sustainable financing have been reported consistently [15]. Non-governmental organisations have played a crucial role in service delivery through outreach and community-based rehabilitation programmes, but reliance on external funding has raised concerns about long-term sustainability [30].

These considerations refer primarily to vision rehabilitation services for adults; the situation is substantially more complex for vision rehabilitation in children and adolescents, particularly in LMICs [15, 31]. Geographic inequities in vision rehabilitation coverage have persisted globally between 2015 and 2025. National surveys documented that a substantial proportion of individuals who could benefit from rehabilitation have never accessed services. In addition to task-shifting approaches, mobile clinics and integration of vision rehabilitation into community-based rehabilitation programmes have shown potential to expand coverage, particularly in resource-constrained settings [15, 30].

### Service Delivery Models and Workforce

As evidence supporting improvements in functional ability is strong, specialised clinic-based vision rehabilitation proved to be the leading model of care [18]. However, the content of these models varies, and they can be resource-intensive and difficult to scale in LMICs. In turn, when vision rehabilitation was integrated into primary eye-care and chronic disease programmes, such as diabetic retinopathy screening, it improved referral and uptake [15].

Workforce shortages, particularly of psychologists, orientation and mobility specialists and occupational therapists trained in low vision, have constrained service expansion globally. Training programmes have been expanded in some regions, but the lack of standardised curricula has remained a barrier [15, 25].

### Innovations in Vision Rehabilitation

In the past decade, telerehabilitation has represented one of the most significant innovations. The COVID-19 pandemic profoundly disrupted in-person rehabilitation services while accelerating the adoption of telehealth and remote models [28]. Systematic reviews and comparative studies indicate that structured telerehabilitation can achieve functional outcomes comparable to clinic-based care for selected patients, particularly when supported by clinicians [32]. In addition, smartphones and tablets increasingly served as rehabilitation platforms, offering built-in accessibility features that improved affordability and reduced stigma [33]. Finally, AI-enabled assistive devices supporting recognition and navigation advanced rapidly between 2015 and 2025 [34].

### Outcomes and Cost-Effectiveness

Evidence collected over more than one decade confirms that vision rehabilitation improves functional ability, independence and vision-related quality of life in people with vision impairment. Benefits have been reported consistently in activities of daily living, mobility, reading performance and psychosocial well-being [18, 32].

Emerging economic analyses suggest that vision rehabilitation is cost-effective when broader societal outcomes are considered, including reduced caregiver burden and improved social, educational and economic participation of people with vision impairment, particularly through increased autonomy, engagement in daily activities and participation in community life [34, 35].

## Achievements in Emerging Fields of Vision Rehabilitation and Technology

Vision rehabilitation is increasingly benefitting from innovations in science and technology, such as remote options for care delivery, AI-based tools for daily use by individuals with vision impairment or interventions that enhance visual input. In the next paragraphs, we present some examples of achievements in emerging fields, such as neuroplasticity and visual system recovery, technological interventions and psychosocial support.

### Neuroplasticity and Visual System Recovery

There is growing interest in the development of vision rehabilitation interventions that optimise or enhance the neural processing of visual input [36–38]. Some interventions, such as perceptual learning, which involves repeated training on a specific visual task [39], rely on residual neuroplasticity within the visual cortex to enable neural processing modifications [40]. Others, such as non-invasive brain stimulation [41, 42], are designed to enhance neuroplasticity and enable remodelling of neural circuits. Different interventions can also be combined in an attempt to enhance neurorehabilitation outcomes [43].

A recent meta-analysis found that perceptual learning significantly improved visual search performance in patients with cortical vision loss [44]. An associated narrative review also identified preliminary evidence that perceptual learning could enhance a range of visual functions, including reading in individuals with low vision. For example, Chung trained a group of six individuals with central vision loss on a rapid serial visual presentation (RSVP) reading task over a 6-week period [45]. A large improvement in RSVP reading speed was observed after training, but this effect did not transfer to other measures of vision, such as visual acuity. A number of questions remain in the field of perceptual learning and low vision, including whether improvements are clinically meaningful, long-lasting and transfer from the trained task to other visual functions [44]. More research is required to explore the potential for clinical translation.

Non-invasive brain stimulation techniques enable the modulation of cortical excitability and neurotransmitter expression in targeted areas of the human brain [46, 47]. Specific techniques include repetitive transcranial magnetic stimulation and transcranial electrical stimulation. There is growing evidence that non-invasive stimulation of the visual cortex can modulate visual perception and enhance visual cortex neuroplasticity [48, 49]. Of particular interest for visual neurorehabilitation, non-invasive brain stimulation can reduce crowding in peripheral vision and enhance contrast sensitivity [48, 50, 51]. Preliminary studies have indicated that non-invasive brain stimulation can enhance peripheral contrast sensitivity in glaucoma [52] and enhance reading of English, but not Chinese characters, in macular degeneration [53]. Non-invasive brain stimulation has also been reported to enhance perceptual learning and reduce visual field defects in small numbers of patients with cortical vision loss [49]. This technology is widely used in other clinical fields such as psychiatry [54] and shows initial promise in the field of vision neurorehabilitation. Larger-scale clinical trials are now required to assess whether non-invasive brain stimulation can truly enhance vision rehabilitation outcomes.

An additional application of perceptual learning in neurorehabilitation under ongoing investigation is the use of prism interventions, particularly for individuals with higher-order visual pathway impairments that present as visuospatial neglect or inattention, or as associated visual midline shifts [55–57]. The pathway damage may occur because of an acquired brain injury resulting from, for example, a cerebrovascular accident. Yoked prism adaptation has shown promise over other methods in assisting with the rehabilitation of individuals with neglect (including an altered subjective straight ahead) [55]. There are also reported alterations in the visual midline resulting from spatial neglect (due to dorsal pathway interruption) [56] or from reduced input to the retinotectal pathway from other systems (such as kinaesthetic and proprioceptive), caused by hemiparesis [57]. These conditions have also been shown to respond to yoked prism application. However, Labreche and colleagues outlined several questions that remain unanswered [58]. Is a visual midline shift part of the neglect compendium or a stand-alone phenomenon? What is the overall efficacy of the prism, and what is the correct amount to offer? Should it be prescribed or used only in therapy? Researchers have created a device to explore these questions further [59]. This novel visual midline gauge uses a moving white target behind a black bar on a black background, allowing controlled horizontal displacement at set speeds with a measurement scale hidden from the participant. This measurement scale enables precise, unbiased assessment of the perceived midline position.

Considering only brain injuries because of a stroke, globally, 12 million people experience a new stroke every year, leaving 94 million people living with its effects [60]. With a 60% incidence of visual impairment post-stroke and of those, a 26.2% incidence of inattention [61], there is heightened awareness of the perceptual phenomenon following medically acquired brain injuries and the need to assess for it, differentiate it from primary visual pathway impairment (hemianopia/quadrantanopia) and if possible, manage it. Moving forward, neurorehabilitation should be recognised as an integral component of low vision rehabilitation to address the needs of patients with visual impairment fully.

### Technological Interventions: Devices, AI and Assistive Technologies

Low vision devices have been used for centuries: a 1629 Rembrandt painting called *Man with a Magnifying Glass* clearly shows a hand magnifier being held for the purpose of improving the user’s near vision. Other than incorporating illumination—first with tungsten bulbs and more recently with LEDs—there have been few significant innovations in hand and stand magnifiers or other optical low vision aids, for many years.

Electronic vision enhancement systems (EVES), such as desktop closed-circuit television magnifiers, were first developed in the 1960s [62]. The principal advantages of these systems over optical magnifiers are their extreme levels of magnification (in some cases, over 100×), their high brightness and their ability to enhance and manipulate contrast (such as increasing the contrast of faint details, or to reverse text polarity such that white print is presented on a dark background) [63]. More recently, portable electronic vision enhancement systems (pEVES) have allowed users to transport these systems more easily. pEVES tend to be preferred over optical magnifiers for leisure reading, and they allow for longer reading duration, but optical magnifiers tend to be more versatile [64].

Wearable EVES (wEVES) have been available for around 30 years [65, 66]. Although wEVES enhance distance and near visual acuity and, in some cases, contrast sensitivity [67], they are still not widely adopted by people with vision impairment. Reasons for this are thought to include their weight, cosmetic appearance, image lag and lack of benefit over existing strategies [68, 69], as well as their high cost. More recently developed wEVES have overcome some of these limitations, although the goal of having a wearable vision enhancement system that looks like a conventional pair of spectacles [70, 71] still has not been achieved. Other wearable devices used by some people with vision impairment include vibrating belts [72] and smart canes [73] for obstacle avoidance.

Many tasks traditionally performed with low vision aids, such as reading a newspaper, dealing with correspondence or checking bank statements, are now performed online [74]. Desktop and laptop computer displays allow text to be displayed at a size, contrast and font type of the user’s choosing. Tablet computers, electronic books and smartphones further allow the display to be held at the preferred viewing position (such as the null point for someone with nystagmus, or the correct working distance for someone using high reading addition spectacles). These systems commonly incorporate text-to-speech technology, allowing people to access information non-visually. Text-to-speech is not only used by people with no perception of light—many people with residual vision prefer to listen rather than view information on their smart devices [33]. Voice assistant systems like Amazon’s Alexa (amazon.com), Google Gemini (google.com) and Apple’s Siri (apple.com) are also beneficial and widely used by people with vision impairment [75, 76].

Many features incorporated in smartphone and tablet computers, such as the camera and zoom function, torch and built-in (or downloaded) magnifier app, are used by people with vision impairment [77, 78]. Navigation apps like Google Maps (google.com) and CityMapper (citymapper.com) provide walking directions and integrate with open public transport data to plan routes and announce the next bus or train to arrive at the user’s location. Navigation apps designed specifically for people with vision impairment include Soundscape (developed by Microsoft: Microsoft.com) and now available as open-source code) and the indoor navigation app GoodMaps (goodmaps.com). Increasingly, people with vision impairment have moved from using specific assistive technology designed for sight loss towards using consumer technology developed for the general population. This move towards universal design, where the needs of people with disability are included at the product design stage, is exciting and very much welcomed.

**Fig. 1:**
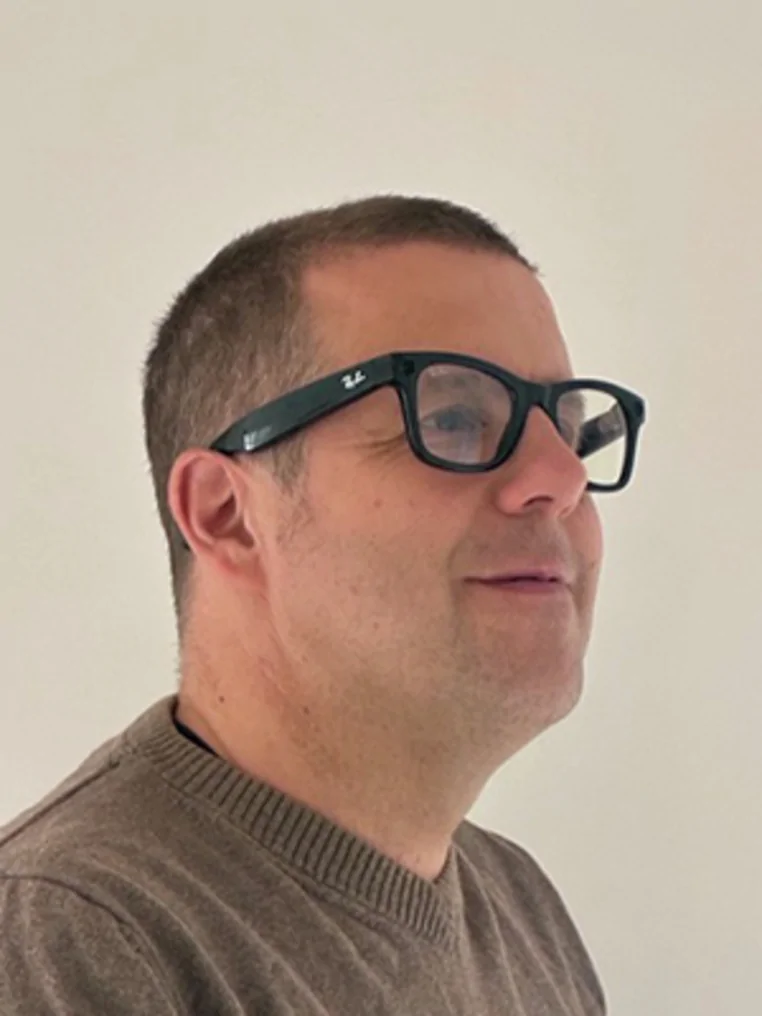
Ray-Ban Meta glasses are being worn by the author MC.

In the past 10 years, AI-based apps have been developed specifically for people with vision impairment. SeeingAI (Microsoft.com) can be used to read text at high levels of accuracy [79] and can also identify faces, colours and banknotes, as well as describing scenes. The AI module of BeMyEyes (bemyeyes.com), an app designed to provide remote human volunteer assistance to people with vision impairment, also provides AI-powered visual scene descriptions, which can be questioned to provide specific details (for example, if the first description of the scene includes a tree, the user can ask the app which type of tree it is, whether it is in leaf and how tall it appears to be). Perhaps most excitingly, AI apps for vision impairment have been included on smart glasses, such as the Ray-Ban Meta (EssilorLuxottica, essilorluxottica.com; Fig. 1), allowing reasonably discreet, ‘always-on’, AI-based assistance for people with low vision in their daily life. First studies show promising evidence in improving task performance, especially when using AI-implementations [80].

### Psychosocial Challenges and Interventions

Irreversible vision loss can profoundly disrupt an individual’s ability to participate in daily life and engage in personally meaningful activities [81, 82]. Adjusting to such a loss often demands substantial psychological, social and practical adaptation [83, 84]. For many individuals, adjustment is an ongoing process that unfolds alongside progressive or irreversible vision loss. Because these losses may resurface as life circumstances change, associated grief may re-emerge. This persistent adjustment process is frequently challenging and is commonly accompanied by psychosocial challenges [83]. Whether people develop mental ill-health seems to be less dependent on the severity and cause of their visual impairment, but more on their coping and adaptive abilities and the social support they receive [83, 84].

The process of adaptation is often difficult, since people with visual impairment face many challenges. Difficulties in performing activities of daily living, such as personal hygiene, housekeeping and transportation use, are common [85]. While people become more dependent on others in performing such activities, their visual impairment may restrict opportunities for social interaction, and they may encounter limited understanding from others [86], contributing to feelings of loneliness [87] and social isolation [88]. People are also less physically active and at greater risk of falling [89]. Moreover, more than half of people with visual impairment report experiencing severe fatigue [90], which is significantly higher than that of individuals with typical sight [91] and may arise from the sustained cognitive effort required to compensate for reduced visual input [92]. Also, poor sleep quality and sleep disorders are frequently experienced by individuals with visual impairment [93].

The biopsychosocial challenges mentioned may increase the risk of mental ill-health. Approximately 25% of individuals with visual impairment report clinically meaningful symptoms of depression [94–96], while 22% report symptoms of anxiety [97]. These rates are significantly higher than those observed in individuals with typical sight [96], indicating that vision loss is a significant risk factor for poor mental health. This is also reflected in a higher tendency toward suicidal ideation [98] and an increased risk of post-traumatic stress disorder [99]. Moreover, while informal caregivers allow people with visual impairment to adapt better, it can take a toll on them, invoking depression and stress [100].

#### Tailored Support and Interventions

The high prevalence of mental ill-health and related challenges, specific to people with visual impairment, emphasises the need for tailored support. Improving early detection is an important first step, since mental ill-health often remains unrecognised due to reluctance among patients and practitioners to discuss symptoms [101]. Several training programmes have been developed, which have improved practitioners’ knowledge and confidence in doing so [102]. Additionally, accessible information for patients and standard screening and referral procedures were found to be important [103].

Some studies found that cognitive behavioural therapy (CBT), in which patients are stimulated to change unhelpful thoughts and behaviours, seems to be promising in reducing depression and anxiety [104–106]. In addition, guided self-help based on CBT has received some attention for its potential to use clinical resources effectively and encourage active change. Kamga et al. found a significant reduction in depressive symptoms by offering CBT-based written and audio tools in addition to minimal telephone guidance by a therapist [107]. Evidence from two other small-scale studies suggests that guided self-help interventions delivered online might be effective in reducing depression and anxiety [108] and severe fatigue [109]. A key strength of these investigations was the adequate accessibility of the online platform that was developed based on a user-centred and iterative approach. For problem-solving interventions, aimed at identifying practical solutions to everyday problems, beneficial effects on vision-related functioning and emotional distress were found, but no long-term evidence to support significant reductions in depression [104, 105, 110, 111]. In addition, several studies indicated possible positive effects on mental health based on counselling, e.g., within low vision rehabilitation services by volunteers and social workers [103].

Peer support may be an additional option to contribute to improved mental health among people with visual impairment [112]. However, while the literature shows positive results on group-based self-management and CBT programmes that incorporate peer support, and a positive effect of peer group emotional support on quality of life was found [113], evidence on the impact of peer support on mental health remains scarce and inconsistent in people with vision loss [103, 104]. To reduce loneliness and social isolation, some qualitative studies found that technology and social media may benefit people with visual impairment; however, there were also several drawbacks and their effectiveness on mental health was not investigated [87].

Several physical activity interventions (e.g., balance training, tai chi, yoga and dance) have been proposed to increase mental health. In a systematic review, these interventions were assessed, but none reported statistically significant improvements in mental health outcomes [89]. Another systematic review looked at the effect of meditation and breathing exercises, which seem to offer a range of benefits for people with vision loss, including improved mood [114]. Interventions for sleep-related problems currently predominantly focus on pharmacological treatments (especially melatonin) while non-pharmacological strategies, like light therapy or CBT, remain underexplored in people with visual impairment [115]. In addition, some evidence for the effectiveness of multicomponent self-management programmes (often group-based) was found [104, 105], where individuals learn to monitor their condition and manage physical and psychosocial consequences by means of psychoeducation, peer support, emotional regulation and behavioural strategies. Finally, a stepped-care programme was found to be effective in improving mental health, sequentially offering four levels of support: watchful waiting, CBT-based guided self-help, problem-solving treatment and, if needed, referral to a general medical practitioner for possible use of psychopharmacological treatment. Patients moved to the next step only when depressive or anxiety symptoms persisted [116].

## Future Directions

### New Standards and Evolution of Multidisciplinary Vision Rehabilitation

Traditionally, vision rehabilitation has been characterised by limited coordination among ophthalmologists, low vision therapists and psychosocial support, often resulting in discontinuity of care [4, 117]. Evidence indicates that the effectiveness of vision rehabilitation depends not only on specific clinical interventions, but also on how services are structured, coordinated and delivered across the lifespan [14, 17]. Integrated, multidisciplinary approaches have been shown to enhance patient outcomes, including improved functional ability and quality of life, compared with fragmented models of care. Future vision rehabilitation services are therefore expected to adopt structured, longitudinal care pathways that support timely referral, coordinated multidisciplinary assessment, shared goals and seamless transitions between clinical and community-based settings. In this perspective, vision rehabilitation is no longer viewed as a short-term or episodic intervention but as an ongoing process that adapts to the evolving needs of individuals, particularly those living with progressive or chronic visual conditions [17, 117].

Future demographic changes highlight the need to redesign collaborative models. By 2030, there will be an estimated 265 million people over 80 years old and by 2070, the population over 65 years is projected to exceed 2.2 billion [118]. Although global age-standardised prevalence of blindness has fallen over the past 30 years due to improved healthcare interventions and prevention programmes, the absolute numbers are projected to rise as populations age [26]. Evidence indicates that older adults have distinct rehabilitation needs, including greater support for falls prevention [119] and mental well-being [120], compared to younger people. Meeting these anticipated needs, coupled with the growing role of assistive technology, will increasingly require collaborative services that extend beyond the traditional remit of optometry-led low vision services to achieve meaningful user outcomes.

Multidisciplinary collaboration remains central to effective vision rehabilitation, but its structure and implementation must evolve to meet changing opportunities and users’ needs. Future models should be evidence-based and responsive to both demographic changes and individuals’ requirements. Strategies including triage, task-shifting, telerehabilitation and establishing wider partnerships will be critical to ensuring effective collaborative care. However, these changes must be accompanied by an input of resources to ensure that a collaborative future for all is achievable and not aspirational.

#### Raising Awareness Across Stakeholders

Limited awareness of the necessity to include vision rehabilitation into care paths among professionals, citizens and policymakers represents a major barrier to the development of vision rehabilitation. Many clinicians still consider rehabilitation as a late or optional intervention, rather than a core component of the eye care continuum. Evidence suggests that early referral to rehabilitation improves functional outcomes and reduces disability-related costs [121]. Awareness initiatives targeting ophthalmologists, orthoptists, optometrists, general medical practitioners and allied health professionals should emphasise the role of rehabilitation alongside clinical treatment.

Public awareness is equally critical. Individuals with visual impairment often remain unaware of available services or believe that irreversible vision loss cannot be addressed. Community education campaigns, collaboration with patient organisations and accessible information materials can promote timely access to rehabilitation and reduce stigma. At the policy level, vision rehabilitation must be explicitly and systematically included in national eye health strategies and universal health coverage frameworks [5], as suggested by the WHO [122].

#### From Evidence to Action: Practical Guidance for Service Implementation

To support scale-up, practical ‘guides for action’ are needed to translate evidence into operational models [123]. At the primary level, vision rehabilitation services should include basic functional vision assessment, prescription of low vision aids, patient education, environmental adaptations and referral pathways to specialised services. In countries where services are limited or absent, a stepwise approach is recommended: identification of service gaps; definition of a minimum rehabilitation package; integration within primary eye care and development of referral and follow-up mechanisms. Strengthening primary-level rehabilitation services may improve equity, reduce travel burdens and ensure continuity of care, particularly for older adults and people living in rural areas.

#### Workforce Training and Capacity Building

A skilled and multidisciplinary workforce is essential for sustainable vision rehabilitation services. Dedicated training programmes are required for ophthalmologists, optometrists, orthoptists, occupational therapists, rehabilitation specialists, psychologists and social workers [123]. Standardised curricula and continuing professional development aligned with international best practices should be promoted. In low-resource settings, task-shifting strategies can help address workforce shortages. Training primary eye care providers and community health workers in basic rehabilitation principles can expand service coverage significantly, if supervision, mentoring and referral systems are in place [124].

#### Personalisation and Needs-Based Service Delivery

Another key direction concerns the increasing personalisation of rehabilitation services. Scientific consensus supports the idea that vision impairment affects individuals differently depending on age, comorbidities, cultural backgrounds, environmental demands and personal goals. Future services are expected to move beyond standardised intervention packages toward needs-based rehabilitation planning. The new ICF Core Set Vision Loss provides a streamlined personalised assessment and guideline for multidisciplinary teams by focusing on the aspects of functioning that are most commonly affected and clinically meaningful, from a consensus-based global perspective [125]. This evolution will involve comprehensive functional assessments that consider vision function, daily activities, participation and quality of life. Rehabilitation goals will be increasingly co-defined with patients and caregivers, also reflecting a shift toward shared decision-making models that are already well established in other areas of healthcare. Such approaches are supported by evidence demonstrating improved adherence and meaningful functional gains [126].

#### Teleassistance and Telerehabilitation

Telerehabilitation has already been shown to provide training across diverse domains of vision rehabilitation, including wearable devices [19] and assistive apps [20], with outcomes comparable to traditional in-person methods. Building on this model, using digital communication platforms could establish geographically dispersed collaborative teams whose reach and expertise extend services beyond existing geographic and knowledge boundaries. Such integration could not only broaden access to multidisciplinary rehabilitation but also facilitate knowledge exchange from higher to lower-resourced countries, supporting capacity-building initiatives. These innovations create opportunities to combine clinical expertise with community-based support, creating hybrid models that blend face-to-face care with remote consultations. Crucially, the successful adoption of this emerging technology must be accompanied by training and infrastructure investment to prevent widening the existing global inequity.

More broadly, telehealth has emerged as a powerful tool to expand access to vision rehabilitation by supporting screening, functional assessment, monitoring and follow-up, particularly for individuals with mobility limitations or living in underserved areas. Remote platforms also enable specialist support for local providers through telementoring and case discussions, fostering capacity building and quality improvement. Advances in digital health technologies for mental health offer further promising possibilities for online interventions [108, 109]. Crucially, the successful adoption of this emerging technology must be accompanied by training and infrastructure investment to prevent widening of the existing global inequity in service provision.

#### Electronic and IT Solutions, Quality Assurance and Outcome Measurement

Electronic health records and digital assessment tools are critical enablers of integrated rehabilitation services. Standardised electronic data collection supports outcome monitoring, service evaluation and health system planning. Future directions emphasise the need for robust quality assurance within vision rehabilitation services. There is growing agreement on the importance of standardised outcome measures that capture functional ability, participation and quality of life [45]. Future services are expected to collect and use core outcome data routinely to guide clinical decision-making, service planning and policy development [127–129]. Aligning such core outcome sets with the ICF Core Set Vision Loss is an important future research direction.

### Future Directions in Emerging Vision Rehabilitation Fields

Vision impairment is no longer considered a static deficit, owing to growing evidence demonstrating residual neuroplasticity in the visual system. This evidence supports the potential for novel intervention methods such as perceptual learning, prism adaptation and non-invasive brain stimulation. Although these approaches are widely explored in research settings, they have not yet been translated into routine clinical practice. Questions regarding clinical feasibility, duration of effects, variability of outcomes and factors influencing response to intervention remain. Integrating research with clinical practice and incorporating neurorehabilitation approaches within comprehensive vision rehabilitation services may help improve real-world rehabilitation outcomes.

Traditional assistive technology has undergone a significant transformation due to rapid technological advances, particularly with the emergence of AI-based applications. Smartphones, wearable devices, navigation tools and AI-assisted scene interpretation have expanded opportunities for independence and participation. They can empower patients to manage daily activities, monitor functional vision and emotional state and communicate with care teams [130]. However, these developments also introduce challenges related to accessibility, usability and safety, particularly for older adults and people with multiple disabilities [130]. Without clinical guidance and structured assessment and intervention plans, technology use may increase cognitive burden or exacerbate existing disparities. This underscores the importance of interdisciplinary collaboration among individuals with vision impairment, clinicians, engineers, designers and data scientists throughout development and implementation.

Psychosocial consequences of vision impairment, including depression, anxiety, fatigue, sleep disturbance, social isolation, as well as caregiver burden, are highly prevalent and have a significant impact on rehabilitation participation and outcomes. While more high-quality studies are needed [104, 105], current evidence supports the effectiveness of targeted psychological interventions, but mental healthcare remains inconsistently integrated into vision rehabilitation services. Rehabilitation models that focus solely on functional vision outcomes often fail to address these psychosocial needs, highlighting an important limitation that must be addressed to achieve truly holistic rehabilitation. Therefore, vision rehabilitation programmes should include territorial psychology and mental health services targeted both to patients and informal caregivers. Psychological assessment, in-person or online counselling, peer support and caregiver education can facilitate adjustment to vision loss, reduce emotional distress and improve adherence to rehabilitation programmes [94, 100].

## Conclusion

This overview demonstrates the increasingly multidisciplinary nature of vision rehabilitation, highlighting how advances in service delivery models, neuroscience, AI, technology and mental health research can contribute to achieving optimal rehabilitation outcomes. Coordinated, individual-centred approaches are essential to address the functional, psychological and social consequences of vision impairment.

Although multidisciplinary models are widely endorsed, their implementation remains challenging due to barriers such as limited resources, workforce capacity and variation in cultural contexts and health policies across regions. Addressing these challenges is essential to improve the availability and effectiveness of multidisciplinary vision rehabilitation services.

Organisations such as ISLRR provide important platforms for interdisciplinary dialogue, bridging basic science, research and clinical practice. By facilitating collaboration among researchers, clinicians, technologists and individuals with lived experience, such organisations can support both research advancement and the implementation of integrated models of care. Together, the evidence reported here points to the need for implementation-focused research, stronger multidisciplinary collaboration and outcome measures that extend beyond impairment to encompass participation, mental health and quality of life. Therefore, embedding multidisciplinary vision rehabilitation within health systems is not only a clinical imperative, but a necessary step toward fulfilling global commitments to inclusion, participation and equity for people with vision impairment.

## Data Availability

No datasets were generated or analysed during the current study.
